# RANKL system in vascular and valve calcification with aging

**DOI:** 10.1186/s41232-016-0016-3

**Published:** 2016-08-01

**Authors:** Ryo Kawakami, Hironori Nakagami, Takahisa Noma, Koji Ohmori, Masakazu Kohno, Ryuichi Morishita

**Affiliations:** 1 Department of Cardiorenal and Cerebrovascular Medicine, Faculty of Medicine, Kagawa University, Kagawa, 761-0793 Japan; 2 Department of Clinical Gene Therapy, Graduate School of Medicine, Osaka University, Kagawa, 565-0871 Japan; 3 Department of Health Development and Medicine, Graduate School of Medicine, Osaka University, 2-2 Yamada-oka, Suita, 565-0871 Osaka Japan

**Keywords:** Vascular calcification, Cardiac valve calcification, Osteoporosis, RANK, RANKL, OPG, VICs, ECs, VSMCs

## Abstract

Vascular and cardiac valve calcification is associated with cardiovascular mortality in the general population. Increasing clinical and experimental evidence suggests that inflammation accelerates the progression of calcification, which has molecules in common with bone metabolism. For example, osteopontin (OPN), osteoprotegerin (OPG), receptor activator of the nuclear factor κB ligand (RANKL), and alkaline phosphatase (ALP) are proposed to play central roles in the calcification or demineralization of atherosclerotic lesions and the calcification of cardiac valves. Abnormalities in the balance of these proteins may lead to perturbations in vascular/valve calcification. “How to prevent calcification” is a common task based on conventional data; however, several pathological findings indicate that heavily calcified plaques are stable, which may not lead to coronary events. Vulnerable plaques tend to be either noncalcified or only mildly or moderately calcified. “How to treat calcification,” which depends on the details of the specific patient, thus remains a difficult challenge. In addition to the detection of calcification, characterization as well as quantification of it is necessary for optimal treatment of this pathology in the future.

## Background

The saying “a man is as old as his arteries” (a person grows old with their blood vessels), which William Osler quoted to describe the association between blood vessels and anti-aging in 1898 [1], remains relevant for many researchers and clinicians who are studying the central concepts of anti-aging. We have realized the importance of these words with the arrival of gluttony and the aging of our society over the past 100 years. For many decades, vascular and cardiac valve calcification has been regarded as consequences of aging. Studies now confirm that vascular and valvular calcification is an actively regulated process and shares many features with bone development and metabolism. Here, we focus on the molecular mechanism underlying the calcification of the aorta and cardiac valves and propose an answer to the question of “how to treat calcification”.

### Bone metabolism and aortic calcification with aging

Aortic calcification is an aging marker during the progression of atherosclerosis. Many studies have reported that aortic calcification and cardiovascular events are highly related in the elderly. This has been demonstrated with the development of recent diagnostic imaging systems. Rodondi et al. prospectively investigated the prognoses of patients aged more than 65 years with vascular calcification for 13 years to determine whether aortic calcification is a risk factor of cardiovascular disease [2]. Patients with aortic calcification were more likely to die of any cause (47 vs. 27 %, *P* < 0.001) and cardiovascular-specific causes (18 vs. 11 %, *P* < 0.001) during follow-up than those without aortic calcification. In analyses adjusted for age and cardiovascular risk factors, aortic calcification was associated with an increased rate of all-cause mortality (hazard ratio (HR), 1.37; 95 % confidence interval (CI), 1.15–1.64) [2]. Okuno et al. followed 515 hemodialysis patients, of whom 291 patients (56.5 %) had abdominal aortic calcification (AAC) [3]. During a mean follow-up of 51 months, there were 103 all-cause deaths, of which 41 were from cardiovascular diseases. Of patients with and without AAC, 27.8 and 9.8 % died, respectively (11.6 and 3.1 % from cardiovascular diseases, respectively). Additionally, using multivariate Cox proportional hazards analysis, the presence of AAC was significantly associated with increased all-cause mortality (HR, 2.07; 95 % CI, 1.21–3.56) and increased cardiovascular mortality (HR, 2.39; 95 % CI, 1.01–5.66) after adjustment for age, hemodialysis duration, diabetes, serum albumin level, and C-reactive protein level. These epidemiologic studies suggest that aortic calcification may be a risk factor as well as a complication.

Vascular calcification and osteoporosis are common age-related processes and are associated with adverse clinical outcomes, including ischemic cardiac events, claudication, and mortality. Vascular calcification was previously considered passive and degenerative, but it is now recognized as a pathobiological process sharing many features with embryonic bone formation. Importantly, several bone matrix proteins are expressed in calcified arteries, which indicate that the cellular and molecular mechanisms of arterial calcification are similar to those of bone metabolism. Bostrom et al. demonstrated the expression of osteogenic differentiation factor bone morphogenetic protein-2 (BMP-2) in calcified human plaques and blood vessel tissue constitutive cells, such as endothelial cells (ECs), vascular smooth muscle cells (VSMCs), and macrophages, in vascular calcification. Furthermore, it was shown that VSMCs could be differentiated into osteoblast-like cells by stimulation with BMP-2 [4]. Both bone metabolic disorder and vascular calcification are degenerative diseases that are common in the elderly population, and they are frequently observed in the same individuals. The constancy of mineral metabolism is maintained by the active balance of osteoblasts and osteoclasts in bone tissue; however, the contribution of osteoclast-like cells to aortic calcification is not yet well understood. Thus, the mineralization processes in bone metabolism and those in vascular calcification may be somewhat different.

### The RANKL system in vascular and valve calcification

**Fig. 1:**
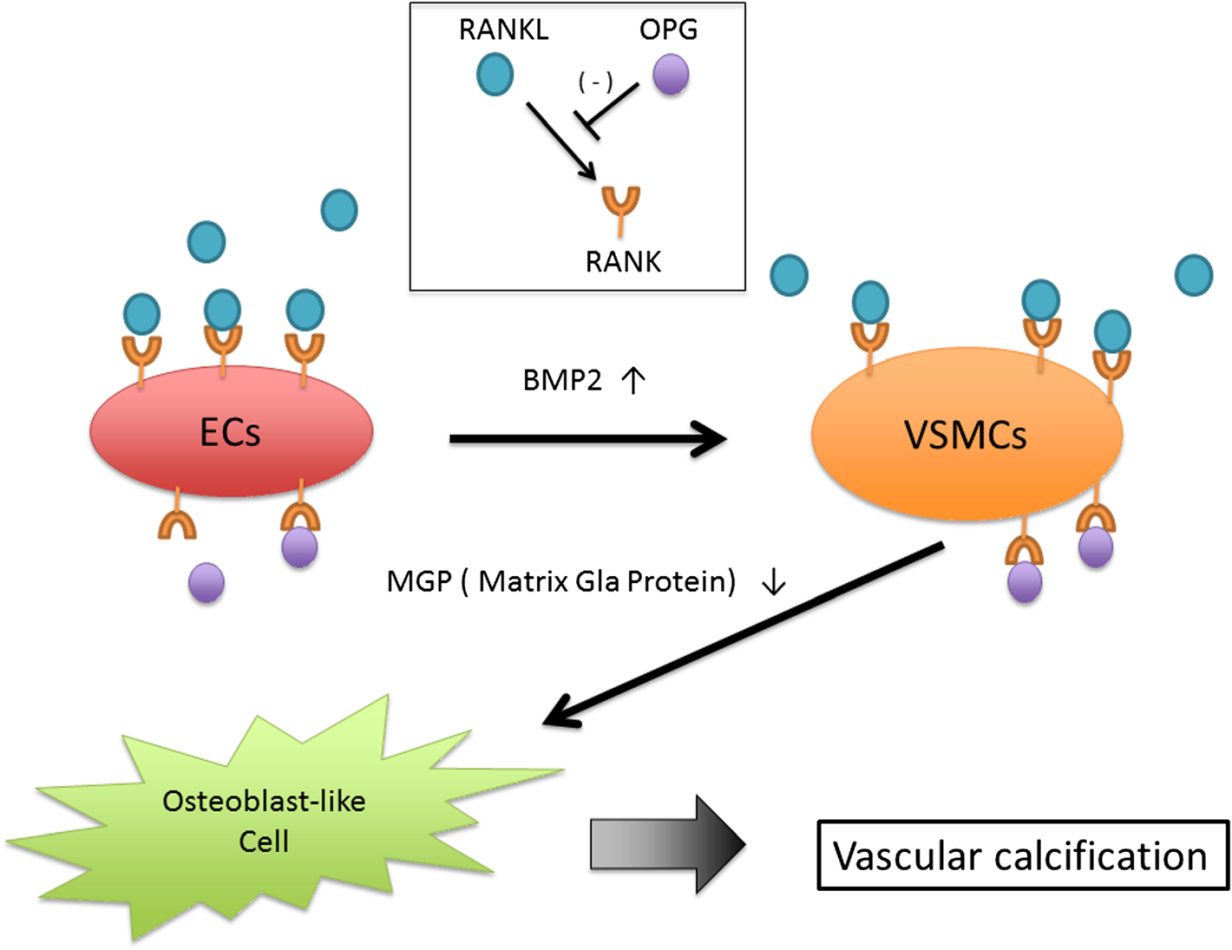
Scheme of vascular calcification through the RANK/RANKL/OPG axis. In vascular cells, RANK is expressed in both ECs and smooth muscle cells (VSMCs), and RANKL is primarily expressed in VSMCs. RANKL directly stimulates osteogenic differentiation of VSMCs via a decrease in MGP and indirectly promotes osteogenesis via BMP2, which is part of the TGF-β superfamily, from ECs. Promoting osteogenic differentiation by RANKL in VSMCs leads to synthesis of bone proteins and matrix calcification within the arterial vessel

To address the unexplored mechanisms for this phenomenon, we focused on the receptor activator of nuclear factor κB (RANK), the RANK ligand (RANKL), and osteoprotegerin (OPG) from the tumor necrosis factor (TNF)-related family, which is associated with this mechanism. RANKL is highly expressed by T cells in lymphoid tissues and osteoblast/stromal cells in trabecular bone, particularly in areas undergoing active bone remodeling or inflammatory osteolysis [5]. In the bone, RANKL binds as a homotrimer to RANK on the surface of monocyte/macrophage lineage cells, and RANKL expression by osteoblasts/stromal cells is essential, together with permissive macrophage colony-stimulating factor (M-CSF) levels, for the complete development of osteoclasts from monocytic precursors under normal or pathological conditions [5]. RANKL activities are blocked by OPG, which functions as a decoy receptor to prevent RANKL/RANK interactions and inhibits osteoclast formation. Interestingly, we demonstrated that the expression of RANKL/RANK/OPG was upregulated in calcified arteries and that RANKL accelerated differentiation of human smooth muscle cells into osteoblast-like cells (Fig. 1). In a human study, serum OPG levels were positively associated with vascular calcification [6]. The diverse actions of RANKL in the bone and the aorta may be the clues to understanding mineralization during aortic calcification.

Aortic valve calcification and stenosis are major medical problems facing an aging society. Calcification of the aortic valve gets increasingly common and occurs in conjunction with high mortality in the setting of advanced age, congestive heart failure, and end-stage renal disease, in which mechanical stress interacts with metabolic and inflammatory disturbances. The identification of osteoblast-like and osteoclast-like cells in human tissue has led to a major paradigm shift in this field. Although valve calcification was considered a passive, degenerative, and untreatable disorder of “wear and tear” unrelated to atherosclerosis, it is now recognized as a disease that is regulated similarly to atherosclerotic calcification, which is promoted by systemic and local inflammatory milieu, characteristic of metabolic syndrome, and type 2 diabetes. Ectopic mineralization of the aortic valve involves several immunological reactions. Accumulating evidence suggests that fibrocalcific remodeling of the aortic valve is associated with activation of the NF-κB pathway and that the expression of TNF-α and IL-6 are increased in mineralized human aortic valves. These activators of the canonical NF-κB pathway promote an osteogenic process, as well as the mineralization of valve interstitial cells (VICs), the main cellular component of the aortic valve.

**Fig. 2:**
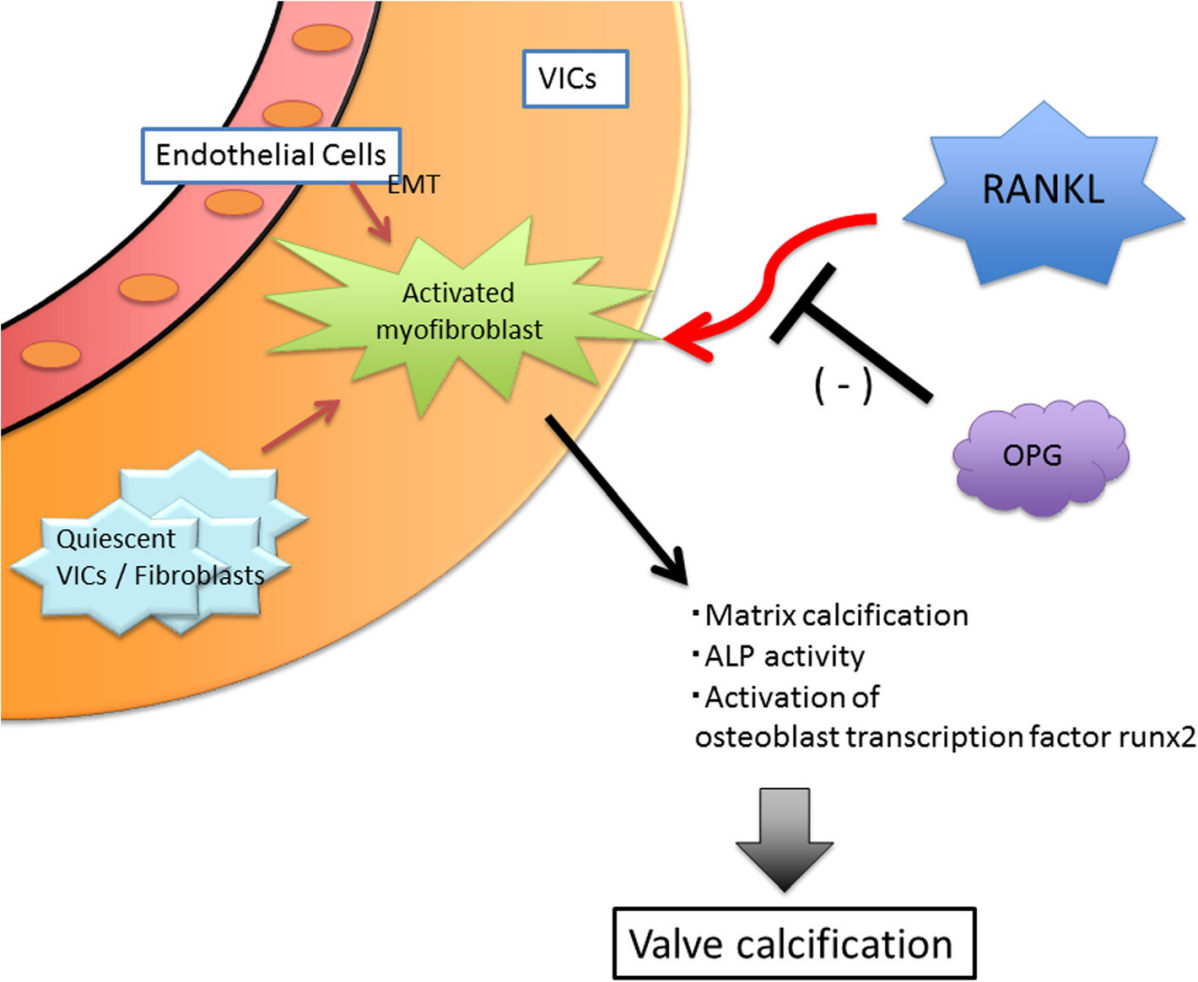
Potential origin of cells that contribute to valve calcification and fibrosis. Valve interstitial cells (VICs) are the main cellular component of the aortic valve. In interstitial cells, activated myofibroblasts are likely to arise from either quiescent VICs or a subpopulation of endothelial cells that undergo endothelial to mesenchymal transformation (EMT). RANKL increased the matrix calcification, ALP activity, and activation of the osteoblast transcription factor runx2 in cultured human aortic valve myofibroblasts. OPG prevents the interaction of RANKL with its receptor, RANK

The RANKL/OPG axis may also regulate aortic valve calcification. Bucay et al. reported that OPG-deficient mice developed osteoporosis and valve calcification [7]. Kaden et al. showed that RANKL was present in human stenotic aortic valves, but not in normal valves. Conversely, OPG expression was higher in normal valves than in stenotic valves [8]. OPG-positive cells were specifically decreased in areas of focal calcification. Moreover, RANKL increased matrix calcification and ALP activity and facilitated the osteoblast transcription factor runx2 in cultured human aortic valve myofibroblasts (Fig. 2). In contrast, Weiss et al. demonstrated that in low-density lipoprotein receptor (LDLR)-deficient ApoB-100 mice fed a high fat diet, the administration of OPG reduced valve calcification through the inhibition of osteogenic transformation but did not prevent valve fibrosis or lipid deposition, suggesting a specific effect of OPG on calcification [9]. These observations suggest that RANKL/OPG may regulate valve calcification both directly and indirectly through regulation of the inflammatory response.

### Therapeutic strategy for calcification

Vascular and valve calcification is an important diagnostic and therapeutic target for the diagnosis and treatment in an aging society. Many studies have reported that vascular and valve calcification is highly associated with mortality. Coronary artery calcium (CAC) has a strong predictive value for incident cardiovascular disease (CVD) events. The Agatston score, the standard CAC score, is weighted upward for greater calcium density. Criqui et al. conducted a multicenter, prospective observational Multi-Ethnic Study of Atherosclerosis (MESA) study at six US field centers with 3398 men and women aged 45 to 84 years [10]. During a median of 7.6 years of follow-up, CAC volume scores showed an independent association with incident coronary heart disease (CHD), with a HR of 1.81 (95 % CI, 1.47–2.23) per standard deviation (SD = 1.6) increase and an absolute risk increase of 6.1 per 1000 person-years, and with CVD, with HR of 1.68 (95 % CI, 1.42–1.98) per SD increase and an absolute risk increase of 7.9 per 1000 person-years. As it progresses, valve calcification can lead to more severe stenosis or regurgitation. Therefore, it is a prognostic factor for cardiovascular mortality. Because the development of calcification should be prevented, several therapeutic drugs to suppress calcified process have recently been described. Allison et al. examined the relationship between estrogen therapy and coronary artery calcium in a randomized clinical trial [11]. They performed computed tomography of the heart in 1064 postmenopausal women who were 50 to 79 years of age at randomization and had undergone hysterectomy. Coronary artery calcium (or Agatston) scores were measured to evaluate calcification. After a mean of 7.4 years of treatment and an additional 1.3 years to be performed (8.7 years after randomization), the mean coronary artery calcium score was lower among women receiving estrogen than among those receiving placebo (83.1 vs. 123.1, *P* = 0.02 by rank test).

As described above, these observations suggest that RANKL signaling may play a permissive role in the development of calcific aortic valve disease. Therefore, vascular and valve calcification has strong relationships with immunity, and both innate and adaptive immunity play a role in the development of calcification. As discussed above, estrogen therapy may have anti-calcific effects. In addition, denosumab, an anti-RANKL monoclonal antibody that is also used for the treatment of osteoporosis, may also exert anti-calcific effects. However, because these therapies have complex biological actions possibly causing side effects, their uses in the prevention of calcification is limited.

**Fig. 3:**
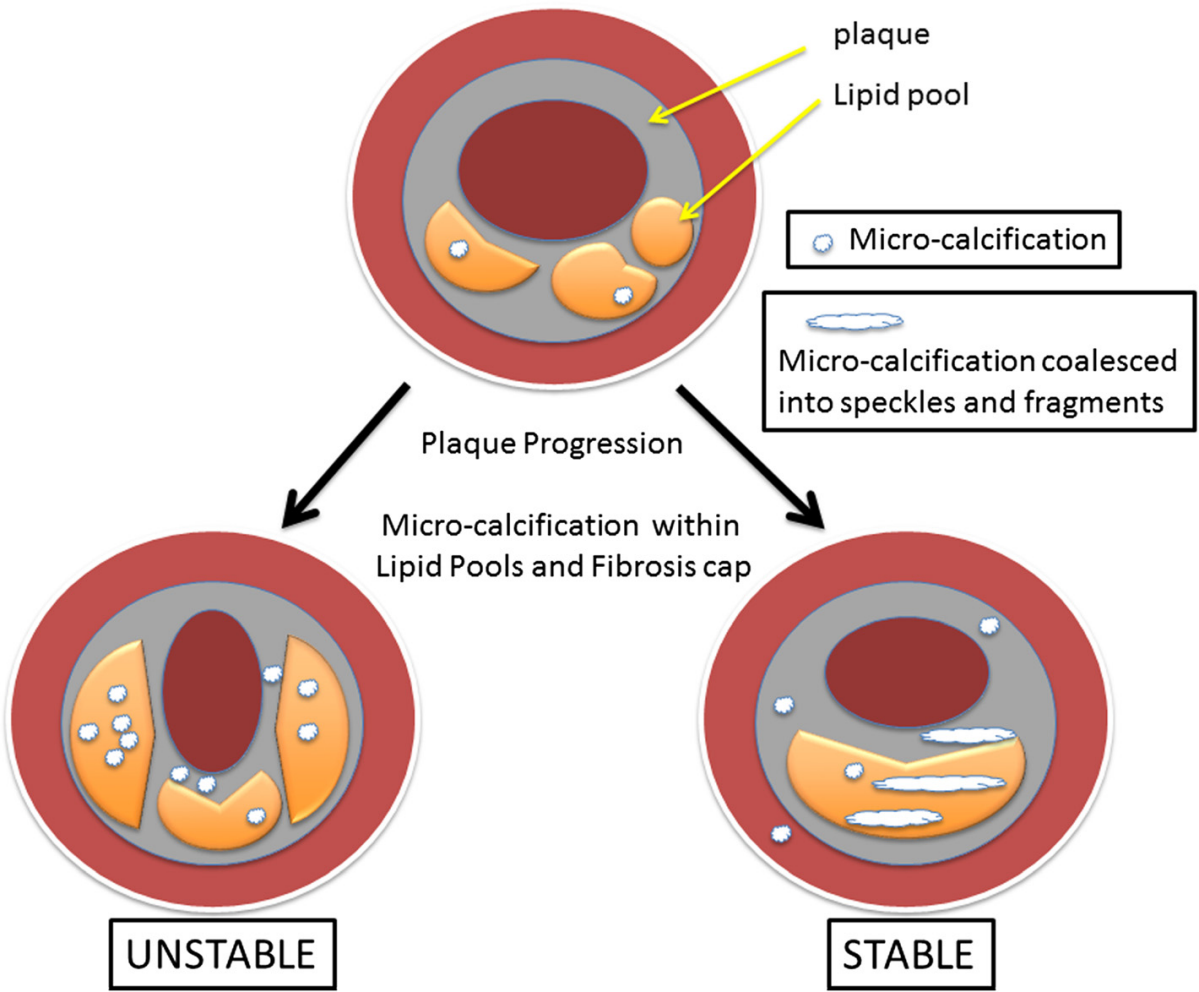
Scheme of plaque calcification. Natural plaque progression involves lipid-pool expansion coupled with micro-calcifications within lipid pools. Micro-calcifications are also commonly found within an overlying fibrous cap. If these micro-calcifications coalesce into speckles and fragments during therapy or atheroma progression, vessel wall stresses may decrease significantly, contributing to plaque stability

We have discussed “how to prevent calcification,” but “how to treat preexisting calcification” remains unclear. We hypothesized that calcification volume itself is responsible to the increased incidence of CVD events. This hypothesis is supported by the observation that CAC volume was positively and independently associated with CHD and CVD risk. Conversely, recent data suggest that increased plaque calcium density may be protective against CVD. Criqui et al. also reported that CAC density scores showed an independent inverse association with CHD, with an HR of 0.73 (95 % CI, 0.58–0.91) per SD (SD = 0.7) increase and an absolute risk decrease of 2.0 per 1000 person-years, as well as CVD, with an HR of 0.71 (95 % CI, 0.60–0.85) per SD increase and an absolute risk decrease 3.4 per 1000 person years [10]. Moreover, Puri et al. reported the contribution of micro-calcification to plaque progression, as shown in Fig. 3. The micro-calcification is commonly observed within an overlying fibrous cap and tends to increase the risk of plaque rupture [12].

At any level of CAC volume, CAC density was inversely and significantly associated with CHD and CVD risk [10]. The role of CAC density should be considered when evaluating current CAC scoring systems. Recently, Hutcheson et al. demonstrated, using three-dimensional collagen hydrogels and high-resolution microscopic and spectroscopic analyses, that calcific mineral formation and maturation results from a series of events involving the aggregation of calcifying extracellular vesicles and the formation of micro-calcifications and ultimately large calcification areas [13]. It is important to evaluate the treatment adaptation of calcification both in vitro and in vivo by utilizing these modalities.

## Conclusions

Vascular and valve calcification has been considered as purely degenerative and unregulated processes until recently. However, growing body of data indicates that there are multiple causes of vascular and valve calcification, including inflammatory, metabolic, genetic, and epigenetic mechanisms, which cross-talk each other in a complicated manner. Although it is important to prevent vascular and valve calcification, it is also important to optimize the therapeutic strategy according to the defined characteristics of present calcification.

## Abbreviations

AAC: abdominal aortic calcification
BMP-2: bone morphogenetic protein-2
CAC: coronary artery calcium
CHD: coronary heart disease
CI: 95 % confidence interval
CVD: cardiovascular disease
ECs: endothelial cells
HR: hazard ratio
M-CSF: macrophage colony-stimulating factor
OPG: osteoprotegerin
RANK: receptor activator of nuclear factor κB
RANKL: RANK ligand
TNF: tumor necrosis factor
VICs: valve interstitial cells
VSMCs: vascular smooth muscle cells
